# First detection of Omicron variant BA.4.1 lineage in dogs, Chile

**DOI:** 10.1080/01652176.2023.2298089

**Published:** 2024-01-04

**Authors:** B. Agüero, F. Berrios, C. Pardo-Roa, N. Ariyama, B. Bennett, RA. Medina, V. Neira

**Affiliations:** a Programa de Doctorado en Ciencias Silvoagropecuarias y Veterinarias, Universidad de Chile, Santiago, Chile; b Departamento de Medicina Preventiva Animal, Facultad de Ciencias Veterinarias y Pecuarias, Universidad de Chile, Santiago, Chile; c Department of Pediatric Infectious Diseases and Immunology, School of Medicine, Pontificia Universidad Católica de Chile, Santiago, Chile; d Department of Child and Adolescent Health, School of Nursing, Pontificia Universidad Católica de Chile, Santiago, Chile; e Department of Pathology and Laboratory Medicine, School of Medicine, Emory Vaccine Center, Emory University, Atlanta, GA, USA; f Department of Microbiology, Icahn School of Medicine at Mount Sinai, New York, NY, USA

**Keywords:** SARS-CoV-2, companion animals, pets, cats, pandemic, one heath

## Abstract

SARS-CoV-2’s rapid global spread caused the declaration of COVID-19 as a pandemic in March 2020. Alongside humans, domestic dogs and cats are also susceptible to infection. However, limited reports on pet infections in Chile prompted a comprehensive study to address this knowledge gap. Between March 2021 and March 2023, the study assessed 65 pets (26 dogs and 39 cats) from 33 COVID-19+ households alongside 700 nasal swabs from animals in households with unknown COVID-19 status. Using RT-PCR, nasal, fecal, and environmental samples were analyzed for the virus. In COVID-19+ households, 6.06% tested positive for SARS-CoV-2, belonging to 3 dogs, indicating human-to-pet transmission. Pets from households with unknown COVID-19 status tested negative for the virus. We obtained 2 SARS-CoV-2 genomes from animals, that belonged to Omicron BA.4.1 variant, marking the first report of pets infected with this lineage globally. Phylogenetic analysis showed these sequences clustered with human sequences collected in Chile during the same period when the BA.4.1 variant was prevalent in the country. The prevalence of SARS-CoV-2 in Chilean pets was relatively low, likely due to the country’s high human vaccination rate. Our study highlights the importance of upholding and strengthening human vaccination strategies to mitigate the risk of interspecies transmission. It underscores the critical role of the One Health approach in addressing emerging zoonotic diseases, calling for further research on infection dynamics and risk factors for a comprehensive understanding.

## Introduction

The severe acute respiratory syndrome coronavirus 2 (SARS-CoV-2) is the causative agent of the COVID-19 pandemic. This enveloped, single-stranded, positive polarity RNA virus belongs to the *Coronaviridae* family, subfamily *Coronavirinae*, genus *Betacoronavirus* (Gorbalenya et al. 2020). Notably, this classification is shared with SARS-CoV-1 and MERS coronavirus, both of which have zoonotic origins (Lee and Hsueh 2020). The genome of SARS-CoV-2 is approximately 30 kb in size, encoding various structural and non-structural proteins, with each playing a distinct role in viral infectivity and transmission. The structural proteins include spike (S), envelope (E), membrane (M), and nucleocapsid (N) proteins (Durmaz et al. 2020; Mittal et al. 2020; Almehdi et al. 2021).

The S protein is a transmembrane glycoprotein, serves as a primary target for neutralizing antibodies due to its receptor-binding function *via* the receptor-binding domain (RBD) (Astuti and Ysrafil 2020; Luan et al. 2020). On the other hand, the N protein, highly immunogenic and abundantly expressed during infection, plays a vital role in RNA transcription, replication, and packaging of the viral genome into virions, while the M and E proteins are essential for virus assembly (Siracusano et al. 2020).

SARS-CoV-2 has demonstrated the ability to infect at least 18 different animal species, encompassing companion animals, captive, farmed, and wild animals (Cui et al. 2022). Among these, the list includes pets such as dogs, cats, ferrets, and hamsters, captive animals including tigers, lions, snow leopards, cougars, lynxes, fishing cats, binturongs, hyenas, otters, coatimundis, hippos, and gorillas, farmed animals such as minks, and wild animals including deer, wild otters, feral minks, and feral cats (Cui et al. 2022).

Surveillance of these cases is of paramount importance to gain a better understanding of the susceptible animal populations and the potential risks associated with the emergence of new hosts and reservoirs. Such reservoirs could serve as potential hiding places for the virus to undergo mutations and possibly re-emerge as new variants that may pose a risk to the human population (CDC 2021). Vigilant monitoring and research in this area are crucial for public health preparedness and to mitigate potential future threats.

Serological prevalence studies have revealed that human-to-companion animal transmission of SARS-CoV-2 in dogs and cats mostly results in cases without clinical signs or mild respiratory signs. However, in a smaller number of cases, these animals can develop disease and show manifestations similar to those observed in humans, including respiratory clinical signs and more severe outcomes, especially when comorbidities are present (Ferasin et al. 2021; Hosie et al. 2021; Keller et al. 2021; Teixeira et al. 2022). Furthermore, the animal detection of the virus in homes with COVID-19 positive individuals has shown varied rates of positivity in pet samples through RT-PCR, ranging from 2.6% to 33% (Sailleau et al. 2020; Calvet et al. 2021; Fuentealba et al. 2021; Jairak et al. 2021; Ruiz-Arrondo et al. 2021).

Global reports of infection cases in dogs and cats are on the rise, as well as surveillance studies focusing on seroprevalence; however, data for South America remains limited. Currently, there are only a few reports of SARS-CoV-2 infections in pets from countries such as Argentina, Brazil, Colombia, Ecuador, Peru, Uruguay, and Chile (Fuentealba et al. 2021; Neira et al. 2021; Schiaffino et al. 2021; Agopian et al. 2022; Alberto-Orlando et al. 2022; Rivero et al. 2022; Panzera et al. 2023). One notable report was published by Neira et al. 2021, detailing a viral detection study conducted in Chile in 2020.

These findings emphasize the importance of monitoring and understanding the potential for SARS-CoV-2 transmission between humans and their companion animals. To contribute to this field, we present a broader study focusing on viral detection in pets from homes under quarantine due to COVID-19. Our investigation spans from the initiation of the human vaccination program in Chile to the end of the pandemic. This research aligns with the position of the WOAH (World Organization for Animal Health), which emphasizes the need for epidemiological investigations of SARS-CoV-2 transmission to and from animals exposed to COVID-19+ patients, targeted surveillance of potential hosts, and continuous monitoring of SARS-CoV-2 mutations in animals. Such efforts are crucial to comprehending viral evolution, the emergence of new variants, and the potential risks they may pose to public health (WOAH 2022).

## Materials and methods

### Design of study and sample collection

Between March 2021 and March 2023, a total of 33 households with at least one confirmed SARS-CoV-2 positive person, as determined by RT-qPCR or antigen test, were recruited for viral detection in this study. As an exclusion criterion, the houses had to have at least one veterinarian in the household, since this person was in charge of taking animal samples during their quarantine period. To conduct the investigation, nasal/oral swabs, fecal samples, and/or environmental samples (food and water bowls gauze) were collected from each animal residing in the COVID19+ households over a period of five consecutive days, starting from the confirmation of the owner’s diagnosis. Nasal/oral swab samples were taken with nasopharyngeal swabs, stool samples corresponded to a fresh environmental stool sample, and environmental samples were taken with sterile gauze soaked in viral transport medium rubbed into dishes of water and/or animal food. All samples were placed in a viral transport medium and appropriately labeled according to individual and date. The study included 26 dogs and 39 cats, comprising 34 females and 31 males, with an age range of 2 months to 15 years. If any animal tested positive for SARS-CoV-2 within the initial five-day sampling period, the sampling was extended for an additional five days. Furthermore, to establish the infection rate in the general animal population, 700 nasal swabs were obtained from animals visiting veterinary clinics. These animals were from households with unknown COVID-19 history and visited the clinics for various reasons, including illness, routine check-ups, or sterilization procedures. The sampling was performed but not limited to the *Región Metropolitana de Santiago* (Metropolitan Region), Chile. The COVID19+ households corresponded to 32 from the metropolitan region and 1 from Los Rios region. The samples of unknown COVID19 status corresponded to 18 animals from Magallanes and Chilean Antarctic region, 69 animals from Bío-Bío region and 613 animals from the Metropolitan region (Figure S1 and Table 1). In this analysis, no information on the socioeconomic aspect of the households was collected.

**Table 1.** Positive RT-PCR samples for SARS-CoV-2 during investigation in 33 COVID-19+ households in Chile, 2021-2023.

Household	Pet	Ct Value	Result	Date	Sample	Age	Sex
20	Dog 15	35,78	Positive	14-04-2022	Environmental	2 years	Female
20	Dog 15	36	Positive	15-04-2022	Oral	2 years	Female
23	Cat 29	37,8	Positive	23-06-2022	Environmental	9 years	Male
27	Dog 21	36,9	Positive	13-07-2022	Nasal swab	7 years	Male
27	Dog 21	36,4	Positive	15-07-2022	Nasal swab	7 years	Male
27	Dog 22	24,6	Positive	11-07-2022	Nasal swab	5 years	Male
27	Dog 22	33,5	Positive	12-07-2022	Nasal swab	5 years	Male
27	Dog 22	34,3	Positive	14-07-2022	Nasal swab	5 years	Male
27	Dog 22	37,5	Positive	15-07-2022	Nasal swab	5 years	Male
28	Dog 23	30,3	Positive	11-07-2022	Environmental	No data	Male

To ensure ethical considerations, all pet owners provided informed consent, and veterinary clinicians collected all samples following the guidelines set by the bioethics committee of the Facultad de Ciencias Veterinarias y Pecuarias of the Universidad de Chile (FAVET, UCHILE) by veterinary clinics (code 21438 – VET – UCH).

### SARS-CoV-2 detection

All samples were tested by real-time RT-qPCR at the Animal Virology Lab at FAVET, UCHILE. Briefly, RNA was purified using Chomczynski-phenol solution (Winkler, BM-1755, Chile). Nasal/oral swab and environmental swab samples were extracted directly from the viral transport solution containing the sample. For fecal samples, a 1:10 dilution of the sample, homogenization, and centrifugation at 2500 g for 10 min were previously carried out. After this step, the previously described RNA extraction was carried out. After this, RT-PCR was carried out to identify a portion of the ORFb1 gene, highly conserved in Sarbecovirus, using the primers *Forward*: 5′-TGGGGYTTTACRGGTAACCT-3′, *Reverse*: 5′-AACRCGCTTAACAAAGCACTC-3′, and 5′-TAGTTGTGATGCWATCATGACTAG-3′ as probe, according to Chu et al. 2020, with a plasmid that contains part of the ORF1b region as positive control. This analysis was performed on a BioRad CFX96 thermal cycler. Samples were considered positive when presented Ct value < 40. Whole genome sequencing was attempted in all positive samples.

### Sequencing and phylogenetic analysis

Positive samples were derived to be sequenced using the ARTIC SARS-CoV-2 ONT (Oxford Nanopore Technologies) sequencing protocol at the Molecular Virology Laboratory at Pontificia Universidad Católica de Chile. The pipeline ARTIC V3/V4 whole-genome amplicon-based sequencing in a minIONTM Sequencer (Oxford Nanopore Technologies) were employed. Briefly, RNA extraction, cDNA synthesis and multiple PCR were done according to Tyson et al. 2020. Then, libraries were performed according to the manufacturer’s instructions using the Native barcodes (EXP-NBD196, and SQK-LSK109). FAST reads were evaluated to determine the quality of the sequencing and then SARS-CoV-2 genomes were assembled even using the modified ARTIC network bioinformatics pipeline (v1.2.1), at https://github.com/artic-network/fieldbioinformatics/issues/59. The clade and lineage were identified according to the Nextstrain and Pangolin nomenclature (Aksamentov et al. 2021; O’Toole et al. 2021). Finally, complete genomes (>95% of coverage) were uploaded to GISAID (Elbe and Buckland-Merrett 2017). Once this information was obtained, the sequences to build the SARS-CoV-2 phylogenetic tree were obtained from a blast search of each sequence obtained, in NCBI and Epicov platforms. Other sequences collected previously from Chilean animals were also included (Neira et al. 2021). Sequence alignment was performed using MUSCLE (3.7) on the CIPRES platform (Miller et al. 2010). The phylogenetic tree was built according to the Randomized Accelerated Maximum Likelihood (RAxML) (Stamatakis 2014) from Geneious Prime software (Biomatters 2023).

### SARS-CoV-2 ELISA

After a minimum of 14 days from initiating the household sampling, serum samples were collected from each animal in the COVID19+ households. The serum samples were tested for SARS-CoV-2 antibodies using the ID Screen® SARS-CoV-2 Double Antigen Multi-species kit (Innovative Diagnostics, Grabels, France) which uses a purified recombinant N antigen covering the wells. Once the samples to be tested and controls are added, anti-SARS-CoV-2 antibodies form an antibody-antigen complex. The peroxidase-labeled purified N protein recombinant antigen conjugate then binds to the free fab of bound anti-SARS-CoV-2 antibodies. This reaction is read at 450 nm and evaluated in S/P%. Values equal to or less than 50% were considered negative, values greater than 50% and less than 60% were considered doubtful, and values equal to or greater than 60% were considered positive, as indicated by the manufacturer. The testing procedure followed the manufacturer’s instructions meticulously. It is crucial to emphasize that cats and dogs can be susceptible to other coronaviruses such as feline coronavirus (FCoV), canine enteric coronavirus (CeCoV), and canine respiratory coronavirus (CRCoV), which lack cross-reactivity with SARS-CoV-2 (Chen et al. 2020; Michelitsch et al. 2020; Patterson et al. 2020; Stevanovic et al. 2021).

## Results

Three dogs from two households (6.06%) tested positive for SARS-CoV-2 by RT-PCR. Additionally, SARS-CoV-2 RNA was detected in environmental samples from three households, with only one household testing positive for both animal and environmental samples. A summary of the positive results can be found in Table 1 and Table 2, and all results of COVID19+ households in Table S1.

**Table 2.** Positive ELISA samples for SARS-CoV-2 during investigation in 33 COVID-19+ households in Chile, 2021-2023.

Household	Pet	S/P%	Result	Date	Sample	Age	Sex
2	Cat 1	278,5237	Positive	25-06-2022	Serum	2 years	Female
8	Dog 5	334,9044	Positive	09-03-2022	Serum	7 years	Male

Among the three dogs positive for RT-PCR, the first is Dog N° 15, a 2-year-old female poodle that does not share the home with other pets. She showed no signs of SARS-CoV-2 infection and tested positive in April 2022, five days after her owner tested positive. However, the animal tested negative to ELISA.

The other two positive dogs belonged to the same household: Dog N° 21, a 7-year-old male giant schnauzer, and Dog N° 22, a 5-year-old male English bulldog. Both showed no signs of SARS-CoV-2 infection and tested positive in July 2022. Dog N° 22 tested negative to ELISA, and Dog N° 21 could not be tested as it passed away. Unfortunately, we were unable to collect additional data and perform a necropsy as the pet owner declined to continue participating in the study. From the detected 3 positive dogs, one of them was positive for a single day, the second was detected positive on two consecutive days, while the third remained positive for a total of 5 days.

In addition to the positive dogs, a 1-year-old female feline (Cat N° 1) and a 7-year-old male canine (Dog N° 5), belonging to two different households, tested positive for SARS-CoV-2 antibodies by ELISA but not by RT-qPCR (Table 2). Overall, antibodies against SARS-CoV-2 were detected in 1 out of 21 cats (4.76%) and 1 out of 17 dogs (5.88%).

The environment-positive samples consisted of gauzes from feeding bowls. One of the samples corresponded to dog N° 15, who was also detected positive for SARS-CoV-2 by RT-qPCR. Another sample was collected from a 9-year-old cat (Cat N° 29) that lives in a household without other pets, and the third sample was from a household with two male dogs, 5 and 7 years old (Dog N° 23 and Dog N° 24). The owners of these households tested positive 5-7 days before the sampling, respectively.

The overall results showed Ct values ranging from 24.6 to 37.5 for the animal samples, while the environmental samples exhibited Ct values ranging from 30.3 to 37.8 (Table 1). On the other hand, all 700 samples obtained from animals in households with unknown COVID-19 status tested negative for SARS-CoV-2 by RT-qPCR.

Out of the total positive samples, four complete SARS-CoV-2 genomes were obtained (Table S3). Two genomes were collected from Dog N° 15, corresponding to one oral swab and one environmental sample. Another genome was obtained from a nasal swab from Dog N° 22, and the last genome was from an environmental sample associated with Cat N° 29. All of these genomes belong into the variant of concern Omicron (B.1.1.529 + BA.*), pangolin BA.4.1 lineage. The sequences obtained are over 29.6 kb in length and have a coverage of more than 213x. They have been submitted to GISAID with the following accession numbers: EPI_ISL_14981147, EPI_ISL_14981148, EPI_ISL_14981149, and EPI_ISL_14981150. The closest sequences found in public repositories, GenBank, and GISAID, to the genomes reported were directly related to human cases in Chile. These sequences were obtained from individuals sampled in the same city (Santiago, Chile) during the same collection year.

**Figure 1.**
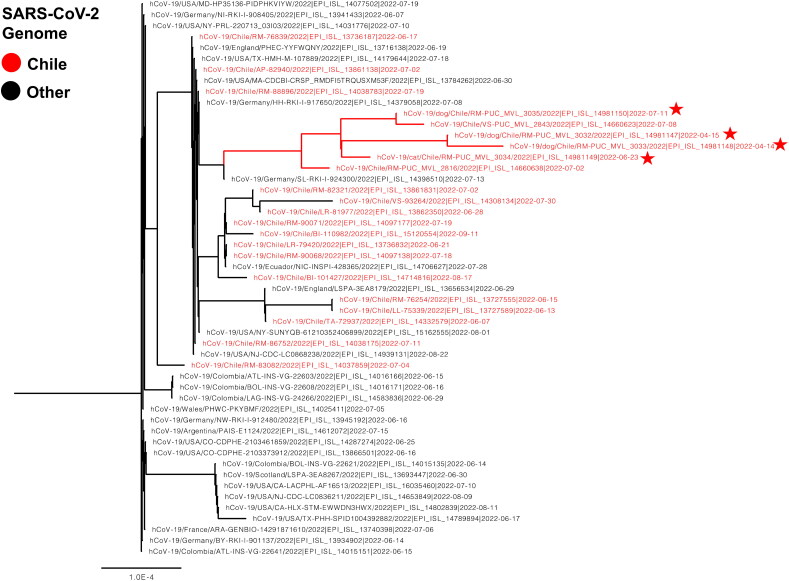
Phylogenetic tree of SARS-CoV2 by using the complete genome. The final dataset included 53 genomes. Chilean sequences are highlighted in red. The animal sequences are depicted with a red star.

The phylogeny reveals that all the sequences obtained in this study belong to the same cluster, which also includes sequences collected from three unrelated humans in the same city and during the same timeframe (Figure 1). It’s worth noting that the older sequences from Chilean cats in 2020 belonged to a different cluster (not shown). Importantly, no other SARS-CoV-2 sequences from animals worldwide demonstrated close genetic relatedness to the sequences obtained in this study.

## Discussion

During the period from March 2021 to March 2023, our study focused on investigating the presence of SARS-CoV-2 in pets from households with COVID-19 positive individuals. Additionally, we examined the general animal population for the presence of the virus. Through this research, we aimed to gain insights into the potential transmission of SARS-CoV-2 between humans and their companion animals, as well as the occurrence of the virus in animals within the broader community.

The obtained results in our study provide confirmation of SARS-CoV-2 infection in pets in Chile, consistent with previous findings reported worldwide. Analyzing the positive RT-PCR SARS-CoV-2 results for animals in Chile during the study period (in COVID19+ households), we observed a general pet positivity rate of 4.62%. This rate is lower compared to the detection rate reported in Chile in 2020 by Neira et al. (2021), who found 11% (3 out of 27 animals) positive for RT-qPCR SARS-CoV-2. Is important to remark, that this study is observational, and the cases were selected conveniently due to the difficulties in recruiting volunteers during the pandemic. As a result, they do not represent a specific neighborhood or social status. Similar difficulties were observed in other investigations around the world.

The lower positivity rate obtained in our research aligns more closely with several studies conducted in 2020, which initially reported very low values of viral detection in animals, ranging between 0% and 5.55% (Chen et al. 2020; Patterson et al. 2020; Sailleau et al. 2020; Sit et al. 2020; Dias et al. 2021; Fuentealba et al. 2021; Goryoka et al. 2021; Hosie et al. 2021; Klaus et al. 2021; Michael et al. 2021; Mohebali et al. 2022; Stranieri et al. 2022).

In contrast, higher detection rates were reported in Brazil, with 27.58% in dogs and 40% in cats (Calvet et al. 2021), in Texas (USA), with 17.6% in cats (Hamer et al. 2021), and in China, with 13.33% in dogs (Sit et al. 2020). Our study contributes valuable data to the understanding of SARS-CoV-2 infection in pets in Chile and provides valuable insights into the variation of detection rates among different geographic regions.

Indeed, studies conducted later in the pandemic have revealed a significant increase in infection rates within COVID19+ households, particularly in felines, with positivity rates ranging from 8% to 35.6% (Alberto-Orlando et al. 2022; Goletic et al. 2022; Meisner et al. 2022; Akhtardanesh et al. 2023; Bae et al. 2023; Hamdy et al. 2023; Kuhlmeier et al. 2023). In dogs, a similar trend is observed, albeit with lower values, varying from 0% to 5% (Meisner et al. 2022; Akhtardanesh et al. 2023), and increasing up to 26.2% (Alberto-Orlando et al. 2022; Goletic et al. 2022; Molini et al. 2022; Bae et al. 2023; Hamdy et al. 2023; Kuhlmeier et al. 2023).

The lower index detected in our study in Chile becomes more pronounced when compared with specific indices reported for other countries in Latin America, such as 24% in Ecuador (Alberto-Orlando et al. 2022), and 30.77% in Brazil (Calvet et al. 2021). Moreover, considering the previous report of 11% in Chile during 2020 (Neira et al. 2021), it is evident that the infection rates have increased over time. These results offsite of Chile could be explained gradual relaxation of control measures associated with COVID-19 cases, which declined in inverse relation to the levels of human vaccination. This relaxation may have facilitated a higher risk of human-to-pet transmission within the same households, making pet contagion more likely. Another possible explanation of the variability of the occurrence of SARS-CoV-2 around the world could be related to the diagnostic method implemented. Most of the studies used real-time RT-PCR, but the targeting gene and the extraction method vary between them, which may implicate differences in sensibility (Table S4).

In contrast to other regions, Chile maintained stricter restricting measures for an extended period and swiftly implemented human vaccination protocols. As a result, Chile stands out as one of the leading democratic countries with remarkably high vaccination rates, a substantial number of vaccine doses administered, a large population of fully vaccinated individuals, and widespread booster administrations. This information is publicly available at Our World in Data (Mathieu et al. 2020). In detail, in Chile, the level of human vaccination has been remarkable, with 93.3% of the population fully vaccinated and 88.9% receiving a booster dose to date, as part of an ongoing vaccination campaign (Mathieu et al. 2020). The vaccination has been associated with decreased viral shedding time and viral load in human populations (Ergoren et al. 2023; Jung et al. 2022). It may have contributed to the lower detection rate of SARS-CoV-2 in pets observed in our study, where a lower overall positivity rate was found in comparison to previous reports from Chile and other countries.

We found a higher positivity rate in dogs compared to cats, which is in contrast to the early pandemic findings suggesting a higher infection rate in cats (Hamer et al. 2021; Krafft et al. 2021). In Chile, a previous report indicated a 17.6% positivity rate in cats and 0% in dogs using RT-qPCR SARS-CoV-2 (Neira et al. 2021). Is well recognized that cats had greater susceptibility of SARS-CoV-2 infection compared to dogs (Bosco-Lauth et al. 2020; Shi et al. 2020). Therefore, these unexpected results should be interpreted with caution, as the number of cases evaluated in our study was limited to 33. This small sample size may not be sufficient to accurately determine positive rates and could only be seen as an additional observation. It’s important to acknowledge that the limited number of COVID19+ cases visited was due to restrictions and concerns for people’s safety during the pandemic, which made it challenging to conduct extensive data collection. Therefore, further studies with larger sample sizes and a more comprehensive approach are necessary to gain a clearer understanding of the prevalence of SARS-CoV-2 in pets from COVID19+ households.

In addition, no SARS-CoV-2 was detected in animals from households with unknown COVID19 status, aligning with research in Turkey (Kadi et al. 2023). The single sampling process without repetitions might have led to an underestimation of the infection in animals in both studies. Similarly, antibodies against SARS-CoV-2 were detected in only 1 cat (4.76%) and 1 dog (5.88%), which is in line with similar findings reported in Portugal and Spain (5% to 7.06%) (Barroso-Arévalo et al. 2022; Oliveira et al. 2022). However, the relatively low rate of seropositivity in cats stands out when compared to other regions. Interestingly, the animals detected as positive by RT-PCR in our study did not exhibit antibodies against the virus after the short viral excretion period, indicating a possible lack of significant infection that triggered the development of immunity (Happi et al. 2023). This situation might be attributed to the animals being initially infected with a low viral load, which could explain the limited immune response.

Other factors related to the variation of the occurrence between countries could include the period of sampling, the variant in circulation during the sampling, the number of households visited, socioeconomic differences, and the time of sampling after human diagnosis. Most of this variability is most likely related to the difficulties of recruitment during the pandemic.

All animal sequences obtained in this study belong to the Omicron variant of concern (B.1.1.529 +BA.*), specifically, the Pangolin lineage BA.4.1. This lineage, BA.4.1, was observed between February 2022 and March 2023 and showed a worldwide distribution. Notably, it was highly frequent in Chilean humans during the same period (Hadfield et al. 2018). The BA.4.1 lineage is a derivative of the BA.4 lineage, which initially originated in Africa and was associated with the fifth wave of infections (Tegally et al. 2022). Currently, there are 68,288 sequenced viruses belonging to the BA.4.1 lineage, with the animal sequences from our study being the only ones reported so far. This highlights the significance of our findings in understanding the transmission and spread of this particular lineage, especially in the context of its presence in animals.

It’s possible that the detected variant is related to the obtained results. The Omicron variant presents 60 unique mutations and has been reported as less severe in humans compared to Delta. Within the Omicron subvariants, BA.4/5 has been reported to be more transmissible and infectious than other Omicron lineages, which could be attributed to its L452R mutation (Pandit and Matthews 2023). This raises the question of whether this mutation could confer an advantage in infecting companion animals, specifically dogs, which could explain the lack of detection in felines in this research, despite substantial evidence of greater susceptibility in cats. Currently, there are few reports on the Omicron variant in pets, and none on the BA.4/5 subvariants. Therefore, there is a pressing need to maintain surveillance of this situation and conduct specific studies to analyze the potential impact of the BA subvariant mutations on animal susceptibility.

It is widely recognized that SARS-CoV-2 can infect animals, and there is substantial evidence of infections with various variants other than BA.4.1(Saied and Metwally 2022; Pandit and Matthews 2023). Interestingly, the USDA (US) reported that so far, most of the WHO variants include the wildtype, *alpha, epsilon, delta, iota, gamma, mu*, and *ómicron*. Notably, the most frequently detected variant in animals has been the delta variant (USDA APHIS 2020). In general, the virus does not transmit well between animals in natural conditions, with the exception of farmed minks (Fenollar et al. 2021) and free-range white-tailed deer (Feng et al. 2023). In fact, SARS-CoV-2 has been successfully disseminated in free-range white-tailed deer, which may serve as a reservoir of extinct VOC (Caserta et al. 2023).

While our study focuses on the BA.4.1 lineage, it is essential to acknowledge the broader context of viral transmission to animals and the potential involvement of different variants in infecting and spreading among them. The coexistence of multiple viral lineages in animals warrants ongoing monitoring and surveillance to better understand the dynamics of interspecies transmission and its implications for public health. In addition, the phylogenetic study indicates that the sequences obtained belong to the human variants of SARS-CoV-2 circulating in the human population during the infection period analyzed. This detection corresponds to its presence in the human population during the year 2022 in Chile, reaching its peak during June 2022 (AN de DM Innovación y Desarrollo (ANDID) 2022), the late autumn season in Chile, consequently with low temperatures as a risk factor for infection in pets, previously published by Murphy et al. 2022. Although the two dogs infected with this variant did not show clinical signs, it is necessary to maintain surveillance under the One Health approach, to early detect mutations or the establishment of new reservoirs that may put public health at risk.

This study confirms the infection of animals by SARS-CoV-2 in Chile in COVID19+ households. However, the values detected are within the lowest range of those detected around the world. This situation can probably be explained by high level of human vaccination in this country. Although more studies are required to confirm this hypothesis, this research suggests this as an additional reason to maintain and strengthen vaccination plans among the human population, under the One Health approach.

## Supplementary Material

Supplemental Material

## Data Availability

The data that support the findings of this study are available in supplementary information, NCBI and Epicov platforms or from the corresponding author upon reasonable request.
